# Disruption of gut integrity and permeability contributes to enteritis in a fish-parasite model: a story told from serum metabolomics

**DOI:** 10.1186/s13071-019-3746-7

**Published:** 2019-10-16

**Authors:** Ariadna Sitjà-Bobadilla, Rubén Gil-Solsona, Itziar Estensoro, M. Carla Piazzon, Juan Antonio Martos-Sitcha, Amparo Picard-Sánchez, Juan Fuentes, Juan Vicente Sancho, Josep A. Calduch-Giner, Félix Hernández, Jaume Pérez-Sánchez

**Affiliations:** 1 Fish Pathology Group, Instituto de Acuicultura Torre de la Sal (IATS-CSIC), 12595 Ribera de Cabanes, Castellón, Spain; 2 Associated Unit of Marine Ecotoxicology (IATS-IUPA), Castellon, Spain; 3 Research Institute for Pesticides and Water (IUPA), University Jaume I, Avda. Vicent Sos Baynat, s/n. Campus del Riu Sec, 12071 Castellón, Spain; 4 Nutrigenomics and Fish Endocrinology Group, Instituto de Acuicultura Torre de la Sal (IATS-CSIC), 12595 Ribera de Cabanes, Castellón, Spain; 5 Department of Biology, Faculty of Marine and Environmental Sciences, Instituto Universitario de Investigación Marina (INMAR), Campus Universitario de Puerto Real, University of Cádiz, 11510 Cádiz, Spain; 6 Comparative Endocrinology and Integrative Biology, CCMar, University of Algarve, Campus de Gambelas, 8005-139 Faro, Portugal

**Keywords:** Myxozoa, *Enteromyxum leei*, Gilthead sea bream, Teleostei, Aquaculture, Pathophysiology, Tight junctions, Gut barrier, Electrophysiology, Metabolomics

## Abstract

**Background:**

In the animal production sector, enteritis is responsible for serious economic losses, and intestinal parasitism is a major stress factor leading to malnutrition and lowered performance and animal production efficiency. The effect of enteric parasites on the gut function of teleost fish, which represent the most ancient bony vertebrates, is far from being understood. The intestinal myxozoan parasite *Enteromyxum leei* dwells between gut epithelial cells and causes severe enteritis in gilthead sea bream (*Sparus aurata*), anorexia, cachexia, growth impairment, reduced marketability and increased mortality.

**Methods:**

This study aimed to outline the gut failure in this fish-parasite model using a multifaceted approach and to find and validate non-lethal serum markers of gut barrier dysfunction. Intestinal integrity was studied in parasitized and non-parasitized fish by immunohistochemistry with specific markers for cellular adhesion (E-cadherin) and tight junctions (Tjp1 and Cldn3) and by functional studies of permeability (oral administration of FITC-dextran) and electrophysiology (Ussing chambers). Serum samples from parasitized and non-parasitized fish were analyzed using non-targeted metabolomics and some significantly altered metabolites were selected to be validated using commercial kits.

**Results:**

The immunodetection of Tjp1 and Cldn3 was significantly lower in the intestine of parasitized fish, while no strong differences were found in E-cadherin. Parasitized fish showed a significant increase in paracellular uptake measured by FITC-dextran detection in serum. Electrophysiology showed a decrease in transepithelial resistance in infected animals, which showed a diarrheic profile. Serum metabolomics revealed 3702 ions, from which the differential expression of 20 identified compounds significantly separated control from infected groups in multivariate analyses. Of these compounds, serum inosine (decreased) and creatine (increased) were identified as relevant and validated with commercial kits.

**Conclusions:**

The results demonstrate the disruption of tight junctions and the loss of gut barrier function, a metabolomic profile of absorption dysfunction and anorexia, which further outline the pathophysiological effects of *E. leei.*
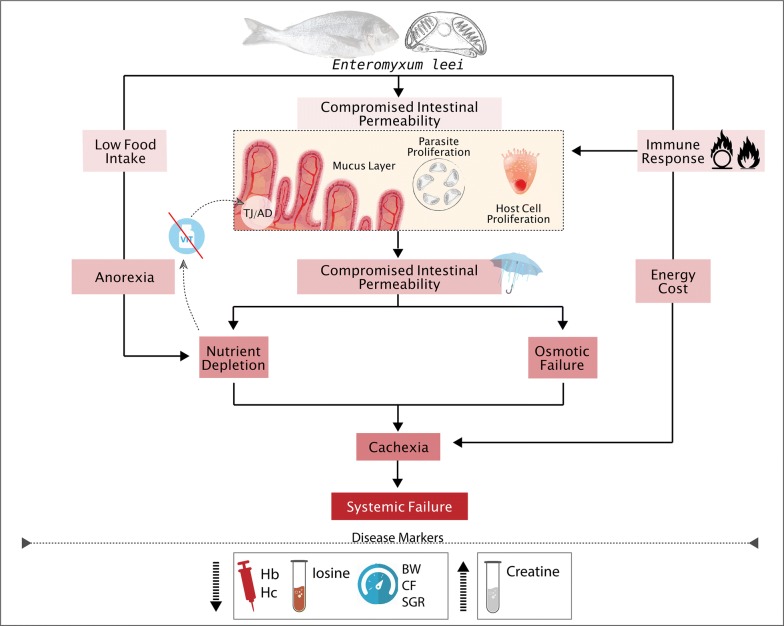

## Background

Enteritis is the inflammation of the intestine in its broader sense. In humans it can be due to viral, bacterial or parasitic infections, induced by exogenous agents (radiation, medication, drug abuse, etc.), or due to inflammatory conditions such as Crohn’s disease or ulcerative colitis. Recent findings also implicate enteric parasites such as *Cryptosporidium parvum* and *Giardia duodenalis* in the development of post-infectious complications such as irritable bowel syndrome and their impact on the neural control of gut functions [1]. In animal production, enteritis is responsible for serious economic losses, intestinal parasitism being a major stress factor leading to malnutrition and lowered performance and production efficiency of livestock and poultry [2]. Furthermore, intestinal health is critically important for welfare and performance in animal production and enteric diseases that cause gut barrier failure result in high economic losses. Common factors in most enteritis scenarios are not only the action of inflammation players, but also the loss of the gut integrity. Intestinal mucus and intercellular tight junctions (TJs) of the epithelial layer act together to maintain the integrity of the gut barrier [3]. The maintenance of the intestinal epithelial barrier is the essential function of the intestinal epithelial cells (IECs), and intraepithelial lymphocytes (IELs) also have sentinel functions in the maintenance of the mucosal barrier integrity [4]. An imbalance in the intestinal barrier structure can flare up into an uncontrollable immune reaction in the intestinal microenvironment or allow the unrestrained growth of microbiota, which leads to various diseases. This loss increases the translocation of bacterial antigens and stimulates inflammation in the intestine [5, 6].

Fish intestine plays various physiological functions that go beyond digestion of food and nutrient absorption. It is also an important immunological site with a key role in protecting the animal from pathogenic insults. Therefore, its integrity is essential to guarantee fish growth, health and welfare [7]. Fish gut integrity has been studied mainly in relation to different dietary interventions that may cause enteritis or several degrees of gut malfunctioning [8–13] and almost no data are available for pathogen-induced enteritis [14]. However, fish intestinal parasitic infections not only cause direct mortalities, but also morbidity, poor growth, higher susceptibility to opportunistic pathogens and lower resistance to stress [15]. The intestinal myxozoan parasite *Enteromyxum leei* dwells between gut epithelial cells and causes severe desquamative enteritis in gilthead sea bream (*Sparus aurata*) (Teleostei), producing anorexia, cachexia, growth impairment, reduced marketability and increased mortality [16]. In advanced *E. leei* infections, the intestine displays hypertrophy of the lamina propria-submucosa and loss of the epithelial palisade structure, together with an intense local inflammatory response [16–19].

Several techniques have been proposed for studying the morphology and physiology of fish gut [20]. However, most of these techniques are time consuming, or expensive and require lethal samplings. In non-piscine hosts, non-lethal markers have been identified to measure gut barrier failure for some enteric pathogens, under field conditions [21]. In humans, several biomarkers have been used to measure gut permeability and loss of barrier integrity in intestinal diseases, but there remains a need to explore their use in assessing the effect of nutritional factors on gut barrier function. Future studies should aim to establish normal ranges of available biomarkers and their predictive value for gut health in human cohorts [22]. Metabolomics are emerging as a valuable tool to find biomarkers in many diseases, as the metabolome includes all small molecules that are present in a biological system and thus, metabolites serve as direct signatures of the metabolic responses and perturbations in metabolic pathways and tightly correlate with a particular phenotype. These properties make the serum metabolome an attractive minimally invasive technique for the identification of system phenotypic perturbations, especially those disruptions due to pathogens [23, 24], and it has started to be used in aquaculture to identify biomarkers indicative of physiological responses of living organisms to environmental or culture conditions [25–27].

The aim of the present study was to outline the gut failure resulting from a well-characterized enteric fish-parasite model using a multifaceted approach (immunocytochemistry, electrophysiology, gut permeability and metabolomics tools) and to find and validate serum non-lethal markers of gut barrier dysfunction. Thus, serum samples from parasitized and non-parasitized fish were first analysed using non-targeted metabolomics and some significantly altered metabolites were selected to be validated using commercial kits with further samples.

## Methods

### Fish infection trials and samplings

**Fig. 1:**
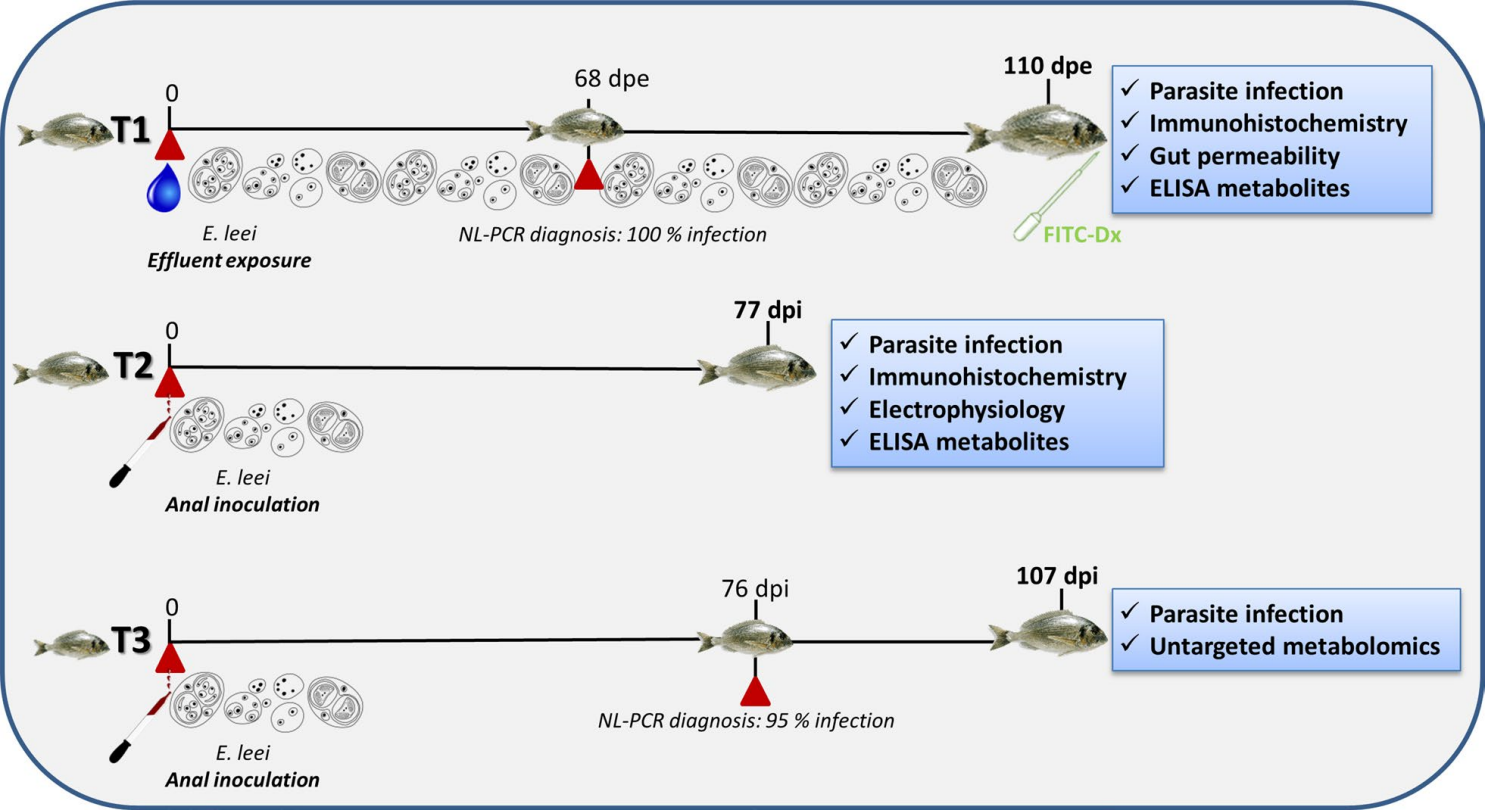
Diagrammatic summary of the different gilthead sea bream infection trials (T) with the parasite *Enteromyxum leei* showing the type of infection, timing, samplings, and the analyses performed at the end of each trial. *Abbreviations*: dpi, days post-inoculation; dpe, days post-exposure; FITC-Dx, intubation with FITC-dextran; NL-PCR, evaluation of the infection by PCR using non-lethal samples

Juvenile specimens of gilthead sea bream (GSB) (*Sparus aurata*) were obtained from commercial fish farms and transported to IATS-CSIC facilities (Castellón, Spain). Before each trial, 20 fish from each stock were sacrificed and checked by qPCR (*18S* ribosomal RNA gene) [28] and histology to be specific pathogen free and clinically healthy. Animals were acclimatized at least 6 weeks before any intervention and were always kept in 5-µm-filtered sea water (37.5‰ salinity), with open flow and natural photoperiod at IATS location (40°5′N, 0°10′E). Temperature was kept constant at 18–19 °C throughout the duration of the trials. Unless stated otherwise, fish were fed *ad libitum* with a commercial diet (EFICO; BioMar, Aarhus, Denmark) throughout all the experiments. Three different trials were performed during this study and are described below. As the parasite dose is not reproducible from one trial to another in this particular model, visual monitoring of clinical signs and non-lethal samplings were performed to evaluate the progression of each infection and select the appropriate timing for a consistent sampling in all trials. The trials are schematically summarized in Fig. 1.

#### Trial 1 (permeability trial)

GSB with an initial weight of 200 g were exposed to *E. leei*-infected effluent as previously described [29] (recipient group, R; *n* = 20) or kept in parasite-free water (control group, C; *n* = 20). They were pit-tagged for individual identification and non-lethally sampled at 68 days post-exposure (dpe) for parasite diagnosis (100% prevalence of infection was detected in the R group). At 110 dpe, C (*n* = 8; mean weight = 410 g) and R (*n* = 8; mean weight = 250 g, with similar infection level at 110 dpe) fish were starved for one day and slightly anesthetized with clove oil (0.1 ml/l) prior to oral intubation with fluorescein isothyocianate (FITC)-dextran (molecular weight 70 kDa; Sigma-Aldrich, St. Louis, MO, USA) in PBS (dosage = 13 mg/kg of body weight). The two experimental groups were held in separate tanks for 5 h to allow intestinal absorption of the permeability marker. Fish were then sacrificed by overexposure to MS-222 (100 mg/ml; Sigma-Aldrich). Blood was taken from the caudal vessels by puncture with heparinized sterile needles and intestinal segments were collected for histological parasite diagnosis. Blood was allowed to clot for 2 h, then immediately centrifuged (15 min, 3000× *g*, 4 °C); the serum was then aliquoted and kept at − 80 °C until analysis.

#### Trial 2 (electrophysiology trial)

One R group of GSB was anally intubated with 0.4 ml of *E. leei*-infected intestinal scrapings, as previously described [30]. Another C group was intubated with PBS (initial fish weight = 97.5 g). Both groups were non-lethally sampled at 76 days post-intubation (dpi) for parasite diagnosis (95% prevalence of infection was detected in the R group). A final sampling was performed at 107 dpi, where 6 heavily infected R fish (average weight = 114.41 g) and 4 C fish (average weight = 222.8 g) were selected by light microscopy observation of intestinal samples obtained by anal cannulation. Serum and histological samples were taken as described before and a portion of anterior intestine was used for the electrophysiology assay.

#### Trial 3 (metabolomics trial)

One R group of GSB (*n* = 25, initial average weight = 213.04 g) was anally intubated with 1 ml of *E. leei*-infected intestinal scrapings, as in trial 2. Prevalence of infection at the non-lethal (NL) sampling (28 dpi) was 100%. A final lethal sampling was done at 77 dpi, in which serum and intestinal samples were taken for metabolomics and histological diagnosis, respectively, from R (*n* = 24, 215.91 g) and C (*n* = 24, 312.54 g) fish.

### Parasite diagnosis

In all trials, parasite diagnosis was performed on anterior (AI) and posterior (PI) intestinal segments fixed in 10% buffered formalin, embedded in paraffin, 4 μm-sectioned and stained with Giemsa following standard procedures. Infection intensity was semiquantitatively evaluated in each intestinal segment using a scale from 1 (lowest) to 6 (highest) as previously described [30]. Non-infected segments were scored as 0. All infected fish had high scores in the posterior intestine, the first segment colonized by this parasite. Based on anterior intestine scores, scores of 1–2, 3–4 and 5–6 were considered low, medium and high infection intensities, respectively. All fish from trials 1 and 2 showed high levels of infection. In trial 3, fish showed different degrees of infection and were grouped accordingly for further analysis.

### Immunohistochemistry (IHC)

In order to evaluate the intestinal damage induced by the parasite, immunohistochemistry was performed using three different markers involved in epithelial integrity: E-cadherin (CDH1), tight junction protein 1 (TJP1 or ZO-1) and claudin-3 (CLDN3). Commercial cross-reacting antibodies were selected for the three molecules, by comparing the sequence of their epitopes with the sequence available in the gilthead sea bream genomic and transcriptomic databases (http://www.nutrigroup-iats.org/seabreamdb/). The selection threshold for the heterologous antibodies was set at 80% of sequence similarity, with long stretches of identical amino acids. In addition, cross-reactivity with undesired proteins was ruled out by blasting the databases.

Four-micrometer-thick sections of anterior, middle and posterior intestine sections from trials 1 and 2 were collected on Super-Frost-plus microscope slides (Menzel-Gläser, Braunschweig, Germany), dried overnight, deparaffinized and hydrated. From each experiment, 4 C and 4 R fish were analyzed. All incubations were performed in a humid chamber at room temperature and washing steps consisted of 5 min immersion in TTBS [20 mM Tris-HCl, 0.5 M NaCl, pH 7.4 (TBS) and 0.05% Tween 20] and 5 min immersion in TBS. Endogenous peroxidase activity was blocked by incubation in hydrogen peroxide 0.3% v/v in methanol (H_2_O_2_:methanol in a 1:9 proportion) for 30 min. Antigen retrieval was performed by boiling the samples in Target Retrieval Solution, pH9 (DAKO, Santa Clara, CA, USA) using a pressure boiler for 30 min. Slides were then washed and blocked 30 min with TBS 1.5% normal goat serum (Vector Laboratories, Burlingame, CA, USA) for the antibodies raised in rabbit (anti-TJP1 and anti-CLDN3) or with TBS 5% BSA for the antibody raised in mouse (anti-CDH1). After washing, slides were incubated with the primary antibodies diluted in TBS 1% BSA for 2 h. The dilutions used were 1:200 for the polyclonal rabbit anti-TJP1 (HPA001636; Sigma-Aldrich) and 1:100 for the polyclonal rabbit anti-CLDN3 (MBS126688; MyBioSource, San Diego, CA, USA). The monoclonal mouse anti-E-cadherin (DAKO, clone NCH-38) was used undiluted and following the protocol previously described [31]. Samples were washed again and incubated with a goat anti-rabbit or a horse anti-mouse antibody (Vector Laboratories) 1:200 in TBS 1.5% normal goat or horse serum, respectively, for 1 h. Slides were subsequently washed and incubated for 30 min with the avidin-biotin-peroxidase complex (ABC, Vector Laboratories), washed and developed by incubating with 3,3′-diaminobenzidine tetrahydrochloride chromogen (DAB; Sigma-Aldrich) for 2 min. The reaction was stopped with deionized water and the slides were counterstained for 2 min with Gill’s haematoxylin before being dehydrated and mounted for light microscopy examination.

### Gut permeability assay

Duplicates of individual sera from R and C fish from trial 1 were diluted 1:1 in PBS, dispensed (100 µl) in 96-well microplates (Thermo Fisher Scientific, Waltham, MA, USA) and read against a standard curve using a range of FITC-dextran concentrations from 2.5 ng/ml to 100 ng/ml. Serum FITC-dextran concentrations were calculated after measuring fluorescence intensity at λem/ex = 535/485 nm in a microplate reader (Tecan Group Ldt., Männedorf, Switzerland).

### Electrophysiology assay

The anterior intestine of C (*n* = 4) and R (*n* = 6) fish from trial 2 was collected, isolated and mounted in Ussing chambers as previously described [32, 33]. Briefly, tissue was washed with chilled saline, opened flat, placed on a tissue holder of 0.71 cm^2^ and positioned between two half-chambers containing 2 ml of physiological saline (NaCl 160 mM; MgSO_4_ 1 mM; NaH_2_PO_4_ 2 mM; CaCl_2_ 1.5 mM; NaHCO_3_ 5 mM; KCl 3 mM; glucose 5.5 mM; HEPES (4-(2-hydroxyethyl)piperazine-1-ethanesulfonic acid, N-(2-hydroxyethyl)piperazine-N′-(2-ethanesulfonic acid) 4 mM), at a pH of 7.8. During the experiments the tissue was bilaterally gassed with 0.3% CO_2_ + 99.7 O_2_ and the temperature maintained at 17 °C. Short-circuit current (Isc, µA/cm^2^) was automatically monitored by clamping epithelia to 0 mV and epithelial resistance (Rt, Ω cm^2^) was manually calculated (Ohm’s law) using the current deflections induced by a 2 mV pulse of 3 s every minute. Voltage clamping and current injections were performed by means of VCC600 or VCCMC2 amplifiers (Physiologic Instruments, San Diego, CA, USA). Bioelectrical parameters for each tissue were manually recorded at 30 min intervals for 150 min after mounting, and data is presented as average of values for each individual.

### Untargeted serum metabolomics

Blood (3 ml) from C and R fish from trial 3 was directly collected into clot activator tubes (BD Vacutainer; BD, Madrid, Spain) and kept on ice for 2 h. After centrifugation (15 min at 3000× *g*, 4 °C), serum samples were aliquoted and stored at − 80 °C until use as described elsewhere [26]. Briefly, one aliquot was deproteinized with acetonitrile for hydrophilic interaction liquid chromatography (HILIC). A second aliquot was evaporated to dryness after acetonitrile deproteinization, and redissolved in methanol 10% for reverse phase (RP) chromatographic analysis. Extracts were then injected in both positive and negative ionization modes (0.7 and 1.5 kV capillary voltages, respectively) in a hybrid quadrupole time-of-flight mass spectrometer (Xevo G2 QTOF; Waters, Manchester, UK) with a cone voltage of 25 V, using nitrogen as both desolvation and nebulizing gas. LC-MS data were processed using the XCMS R package (https://xcmsonline.scripps.edu) with Centwave algorithm for peak picking (peak width from 5 to 20 s, S/N ratio higher than 10 and mass tolerance of 15 ppm), followed by retention time alignment, peak area normalization (mean centering), log 2 applying (to avoid heteroscedasticity) and Pareto scaling. For elucidation purposes, fragmentation spectra of features of interest were compared with reference spectra databases (METLIN, http://metlin.scripps.edu; Human Metabolome DataBase, http://www.hmbd.ca; MassBank, http://www.massbank.eu). For unassigned metabolites, *in silico* fragmentation software (MetFrag, http://msbi.ipb-halle.de/MetFrag), with subsequent searches through Chemspider (http://www.chemspider.com) and PubChem (https://pubchem.ncbi.nlm.nih.gov) chemical databases, was employed.

### Targeted metabolite detection in serum samples

The concentration of creatine and inosine were measured in serum samples of C and R fish from trials 1 and 2 using specific kits. These two metabolites were selected due to the availability of commercial kits to measure their concentration in serum samples and their significant differential abundance and presence among the VIP variables from the untargeted metabolomics study (see below). Creatine was measured with the Creatine Assay Kit (KA1666; Abnova, Heidelberg, Germany) using 10 µl of each serum sample in duplicate following the manufacturer’s instructions. A calibration curve ranging from 0.5 to 50 µM of creatine was included in the assay and the concentration in each sample was extrapolated after measuring fluorescence intensity at λem/ex = 590/530 nm. Inosine was measured using an Inosine Assay Kit (MAK100; Sigma-Aldrich) using 5 µl of each serum sample in duplicate, following the manufacturer’s instructions. A calibration curve ranging from 0.1 to 0.5 nmol/well was included in each assay and the presence of inosine was determined measuring the fluorescence intensity at λem/ex = 590/530 nm.

### Statistics and data analyses

Data from the electrophysiology, gut permeability assays and metabolite detection by ELISA were analysed for statistically significant differences between C and R groups by Student’s t-test or the Mann–Whitney test when Shapiro–Wilk normality test failed, using SigmaPlot v.13.0 (Systat Software, San Jose, CA, USA). Differences were considered significant at *P* < 0.05. To study the separation among experimental groups, partial least-squares discriminant analysis (PLS-DA) was performed using EZinfo v.3.0 (Umetrics, Umeå, Sweden). The quality of the PLS-DA model was evaluated by the parameters R2Y(cum) and Q2Y(cum), which indicate the fit and prediction ability, respectively. To discard the possibility of over-fitting of the supervised model, a validation test consisting in 999 random permutations was performed using SIMCA-P+ v.11.0 (Umetrics). The contribution of the different metabolites to the group separation was determined by variable importance in projection (VIP) measurements. A VIP score > 1 was considered to be an adequate threshold to determine discriminant variables in the PLS-DA model [34, 35].

## Results

### Tight junction protein 1 and claudin 3 protein expression is affected by *E. leei*

**Fig. 2:**
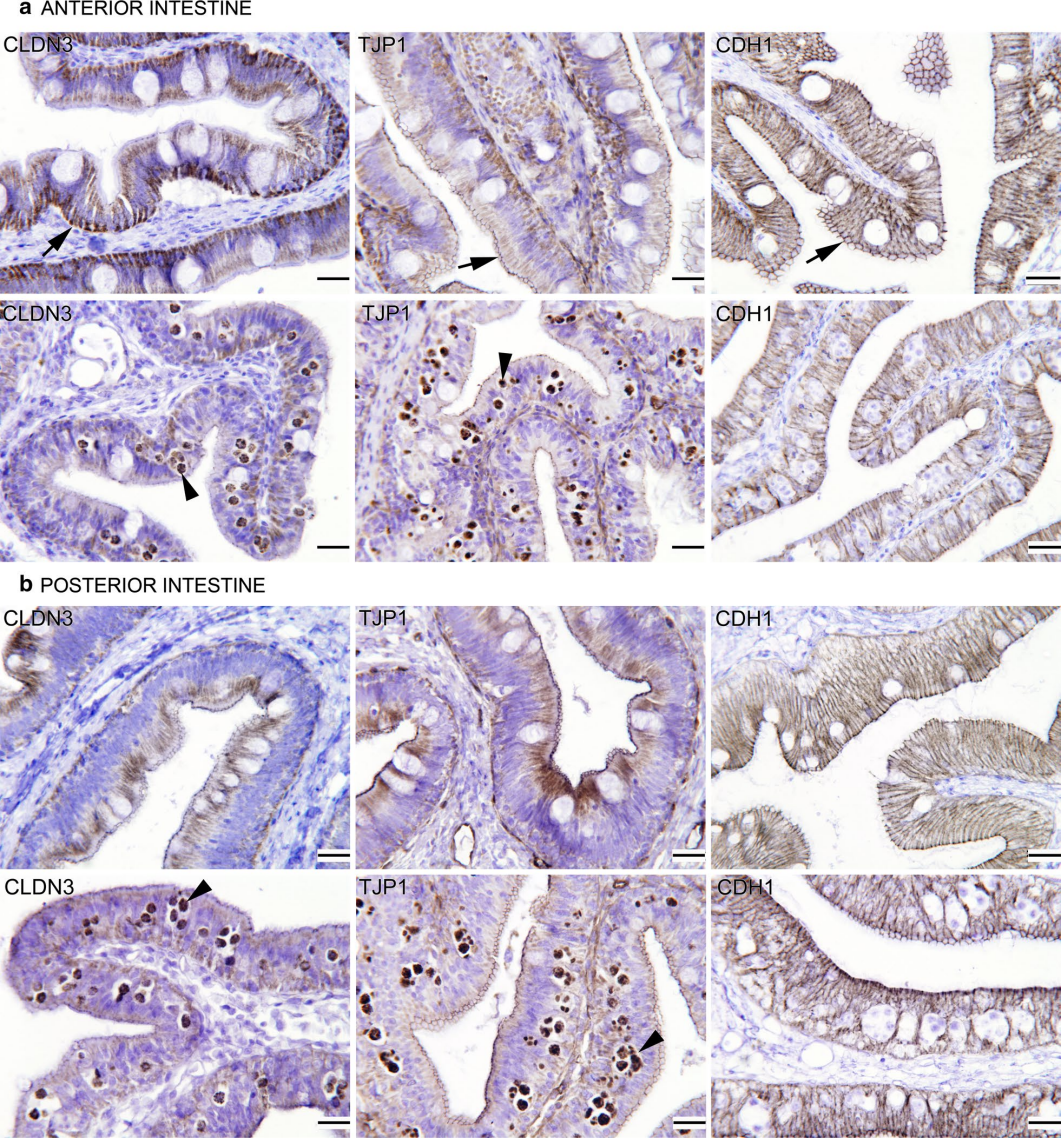
Photomicrographs of gilthead sea bream sections of anterior (**a**) and posterior (**b**) intestines immunolabelled (brownish colour) with antibodies against claudin 3 (CLDN3, left pictures), tight junction protein 1 (TJP1, central pictures) and E-cadherin (CDH1, right pictures). For each intestinal segment, the upper panel corresponds to control healthy fish and the lower panel to *Enteromyxum leei*-parasitized fish. Arrowheads point to some labelled parasitic stages, and arrows to some of the positive immunostaining of control fish at the anterior intestine. Note the differences in the distribution and staining intensity in parasitized intestinal sections*. Scale-bars*: 20 µm

CLDN3 is an integral membrane protein component of TJ proteins, contributing to create an ion-selective border between apical and basolateral compartments. Thus, as expected, the anti-CLDN3 antibody marked strongly the basal membrane of the intestinal epithelium and the lateral membranes of enterocytes in the three intestinal segments of control fish, although it was stronger at the AI (Fig. 2a, left pictures). By contrast, the immunolabelling decreased in parasitized intestines (in all intestinal segments), particularly at the lateral junctions at the PI (Fig. 2b, left pictures).

TJP1 is an important intracellular TJ protein, linking the cell cytoskeleton to the transmembrane TJ proteins. The anti-TJP1 antibody marked strongly the basal membrane and the apical epithelium, with a dot-lined style, in all intestinal segments of control animals, being higher at the AI (Fig. 2a, middle pictures). In parasitized fish, however, the immunolabelling was not so strong and decreased similarly in all the sites. It is remarkable that some parasitic stages (secondary and tertiary cells) were also strongly labelled with this antibody (Fig. 2a, b, middle pictures).

CDH1 is a transmembrane protein that acts as a cell adhesion molecule, important in the formation of adherens junctions to bind cells with each other. The anti-CDH1 antibody stained similarly the lateral junction of enterocytes in all intestinal segments of control fish, and the labelling hardly changed in parasitized fish (Fig. 2a, b, right pictures).

### Parasitized fish showed an increased intestinal permeability

**Fig. 3:**
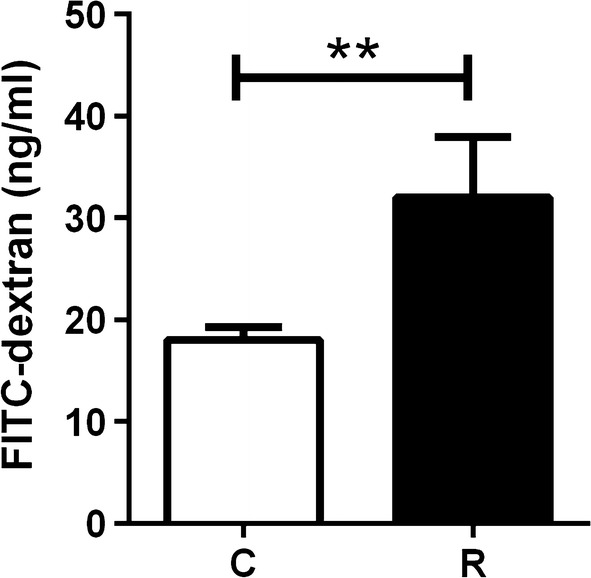
Gut permeability increases in *E. leei* infected fish. FITC-dextran concentration (ng/ml) in serum from control (C, *n* = 8) and recipient (R, *n* = 8) fish 5 h after oral intubation with 13 mg/kg of FITC-dextran. Data are presented as mean + SEM. Asterisks (**) denote statistical significance at *P* < 0.01 (Mann-Whitney test, *P* = 0.0047)

The paracellular transport of small macromolecules across the intestinal epithelium was assessed through the translocation of 70 kDa FITC-dextran into the blood stream. The FITC-dextran concentration in blood serum of R fish was significantly higher than in C fish (Mann-Whitney U-test: *U*_(8)_ = 6, *Z* = − 2.83, *P* = 0.0047) (Fig. 3). All R fish used for this analysis were infected at the three intestinal segments with high infection intensity.

### Intestinal transepithelial resistance is lower in parasitized fish

**Fig. 4:**
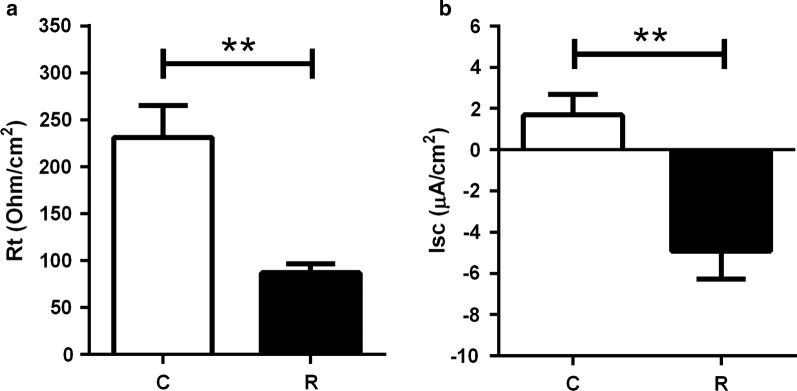
Intestinal tissue integrity and absorptive function are impaired in *E. leei* parasitized fish. Electrophysiology results showing **a** transepithelial electrical resistance (Rt, Ω.cm^2^) and **b** short circuit current (Isc, µA/cm^2^) of control (C, *n* = 4) and recipient (R, *n* = 6) fish anterior intestines. The data represent the mean (+ SEM) of the tissue Rt or Isc values along the 150 min of *ex vivo* experiment with the Ussing chambers. Asterisks (**) denote statistical significance at *P* < 0.01 (**a** Mann-Whitney test, *P* = 0.0095; **b** Student’s t-test, *P* = 0.0042)

Rt (Ω cm^2^), a measure of tissue integrity, was monitored for each AI *ex vivo*. In C fish, Rt steadily raised until 90 min after mounting, as expected, and remained stable thereafter. However, in R fish Rt values remained low and stable throughout the testing time (data not shown). The mean Rt values of the stabilized measurements were significantly higher in C than in R fish (Mann-Whitney U-test: *U*_(4)_ = 24, *Z* = 2.59, *P* = 0.0095) (Fig. 4a). In addition, short circuit current (Isc, μA/cm^2^) was also recorded for each epithelial preparation (t-test: *t*_(8)_ = 3.95, *P* = 0.0042) (Fig. 4b). Under the current experimental conditions, positive Isc values are associated with absorptive function as it was detected in C fish, whereas the negative Isc values found in R fish indicate a secretory function, reflecting the prevailing electrolyte transport across the epithelium. Thus, C fish exhibited an absorptive (positive) current that reflects a proper function of the epithelium, whereas infection induced a persistent and non-reversed secretory current throughout the measuring period reflecting an *in vivo* persistent diarrhea (negative mean values for R group).

### Parasitized fish show significant changes in their serum metabolomics profile

**Fig. 5:**
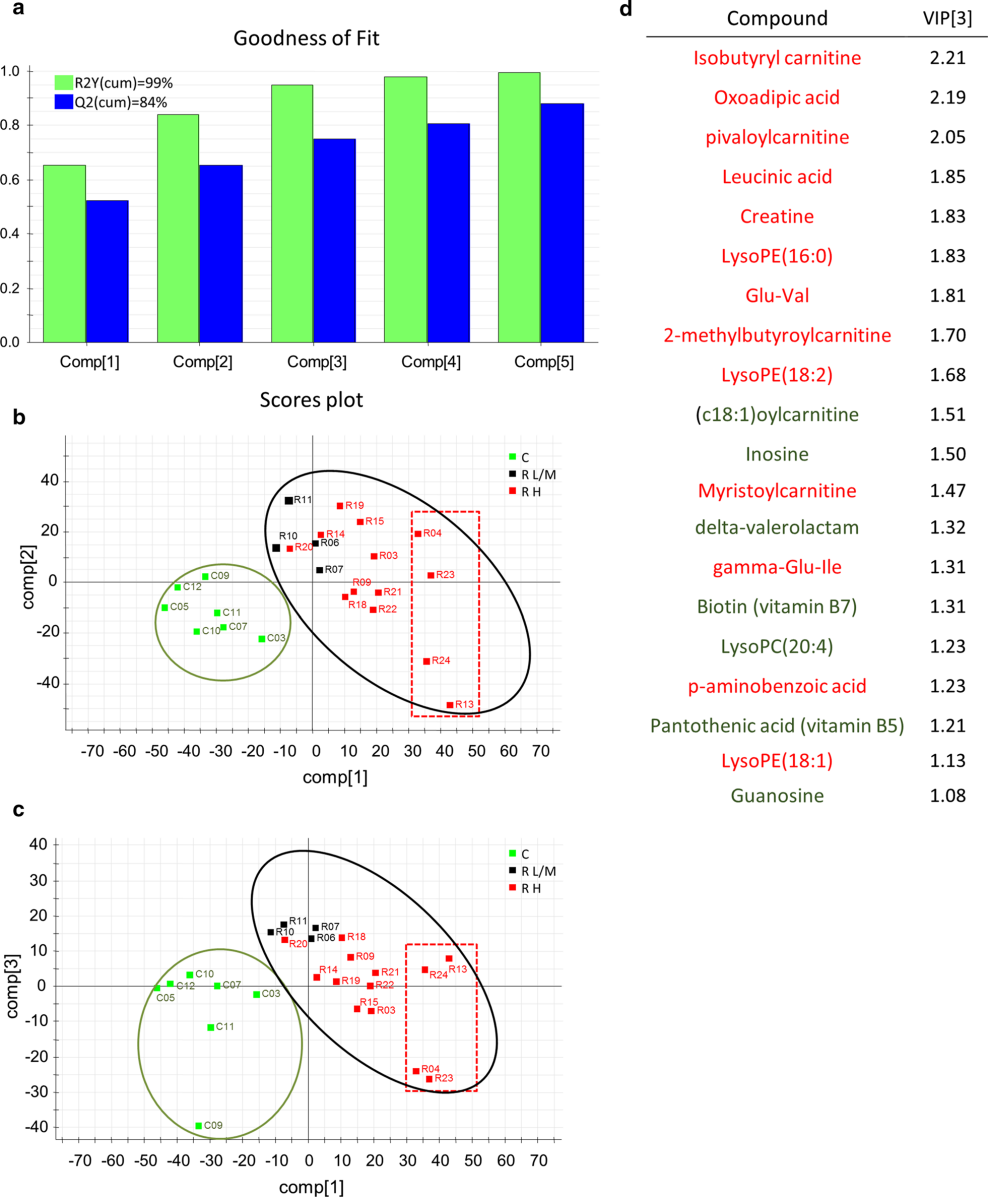
PLS-DA analysis of serum metabolomics. **a** Graphical representation of the goodness-of-fit. The three first components explained more than 90% and predicted more than 75% of the variance. **b**, **c** PLS-DA score plots representing the distribution of samples with component 1 *vs* component 2 (**b**), and component 1 *vs* component 3 (**c**). All infected recipient (R) fish clustered separated from control (C) fish. In addition, R fish with high intensity of infection (H) were more separated from C than R with low (L) and medium (M) infection levels. R fish with the highest infection levels are included in the rectangle. The contribution of the different metabolites to the group separation was determined by variable importance in projection (VIP) measurements after three components. **d** List of the metabolites increased (in red) or decreased (in green) during the infection, and their VIP (variable importance in projection) scores

A total of 3702 ions were detected in all four injections (reversed phase and HILIC chromatographies in both positive and negative ionization modes). Among them, 182 showed a *P* (corrected) higher than 0.5 in a OPLS-DA statistical method, so they were selected for further study (Additional file 1: Figure S1). Some of them showed differences between molecular ion isotopes of 0.5, 0.33 or 0.25 mDa, which were considered peptides or protein fragments with more than a single charge. However, their small intensity made their identification by means of tandem MS really difficult, hampering their final elucidation. Other compounds highlighted by OPLS-DA were studied in MS/MS experiments at 10, 20, 30 and 40 eV collision energy, obtaining a list of 20 tentatively elucidated compounds (Table 1), related to different biological processes [fatty acid oxidation (5 compounds), amino acid catabolism (4 compounds), energy homeostasis (1 compounds), nucleoside metabolism (2 compounds), lysophospholid metabolism (4 compounds) and vitamins and polyphenols metabolism (4 compounds)]. The differential expression of these 20 identified compounds markedly separated control from infected groups in multivariate analyses (PLS-DA), in which the three first components explained more than 90% and predicted more than 75% of the variance. This analysis separated also R groups by low/medium and high intensity of infection (Fig. 5), although the statistical significance of the prediction was restricted by the number of fish in each R group category.

**Table 1:** Highlighted (↑, upregulated; ↓, downregulated) compounds obtained from untargeted metabolomics of serum samples of gilthead sea bream inoculated with *Enteromyxum leei*. Non-infected (C) fish were compared with highly (R-H) or low/moderately (R-L/M) infected recipient (R) fish

Compound	Biological process^#^	Feature name	Chromatography/ ionization mode	Formula	m/z (Da)	R-H, % C	R-L/M, % C	Corrected *P*-value*
Isobutyryl carnitine ↑	1	M232T96_RPPOS	RP/+	C_11_H_21_NO_4_	232.2967	1615^b^	969^c^	6.78E^−3^
Pivaloylcarnitine ↑	1	M246T150_RPPOS	RP/+	C_12_H_23_NO_4_	245.3153	1128^b^	664^c^	5.72E^−3^
2-methylbutyroylcarnitine ↑	1	M246T155_RPPOS	RP/+	C_12_H_23_NO_4_	246.3233	955^b^	470^c^	1.42E^−2^
Myristoylcarnitine ↑	1	M372T767_RPPOS	RP/+	C_21_H_41_NO_4_	372.5625	303^b^	127^a^	3.37E^−2^
(c18:1)oylcarnitine ↓	1	M426T788_RPPOS	RP/+	C_25_H_47_NO_4_	426.3571	32^b^	36^b^	6.21E^−03^
Oxoadipic acid ↑	2	M159T74_RPNEG	RP/−	C_6_H_8_O_5_	159.1168	4704^b^	8660^c^	1.63E^−2^
Leucinic acid ↑	2	M131T254_RPNEG	RP/−	C_6_H_12_O_3_	131.1497	949^b^	632^c^	1.39E^−2^
γ -Glu-Val ↑	2	M247T94_RPPOS	RP/+	C_10_H_18_N_2_O_5_	247.2683	402^b^	202^c^	7.33E^−3^
γ-Glu-Ile ↑	2	M261T171_RPPOS	RP/+	C_11_H_20_N_2_O_5_	259.2790	343^b^	255^b^	2.60E^−2^
Creatine ↑	3	M132T73_RPPOS	RP/+	C_4_H_9_N_3_O_2_	132.1411	425^b^	245^c^	1.26E^−2^
Inosine ↓	4	M267T72_RPNEG	RP/−	C_10_H_12_N_4_O	203.2205	94^a^	77^b^	2.37E^−2^
Guanosine ↓	4	M282T73_RPNEG	RP/−	C_10_H_13_N_5_O_5_	282.2328	70^a^	58^b^	4.42E^−2^
LysoPC(20:4) ↓	5	M544T874_RPPOS	RP/+	C_28_H_50_NO_7_P	543.6729	67^b^	46^b^	6.13E^−2^
LysoPE(18:2) ↑	5	M478T906_RPNEG	RP/−	C_23_H_44_NO_7_P	476.5638	199^b^	106^a^	1.31E^−2^
LysoPE(16:0) ↑	5	M452T895_RPNEG	RP/−	C_21_H_44_NO_7_P	454.5583	259^b^	143^b^	4.05E^−3^
LysoPE(18:1) ↑	5	M480T944_RPNEG	RP/−	C_23_H_46_NO_7_P	480.5855	185^b^	123^a^	2.14E^−2^
Biotin (vitamin B7) ↓	6	M245T223_RPPOS	RP/+	C_10_H_16_N_2_O_3_S	245.3186	28^b^	66^c^	2.69E^−2^
Pantothenic acid (vitamin B5)↓	6	M220T115_RPPOS	RP/+	C_9_H_17_NO_5_	220.2429	27^b^	71^c^	3.56E^−2^
Delta-valerolactam ↓	6	M100T100_RPPOS	RP/+	C_5_H_9_NO	100.1390	16^b^	38^c^	6.72E^−3^
p-aminobenzoic acid (PABA) ↑	6	M138T94_RPPOS	RP/+	C_7_H_7_NO_2_	137.0474	177^b^	215^b^	1.44E^−2^

^#^1, fatty acid oxidation; 2, amino acid catabolism; 3, energy homeostasis; 4, nucleoside metabolism; 5, lysophospholid metabolism; 6, vitamins and polyphenols metabolism

*Benjamini-Hochberg multiple testing correction

*Note*: Superscript letters indicate statistically significant differences, using letter ^a^ for C group

### Inosine and creatine are good serum markers of parasitized fish

**Fig. 6:**
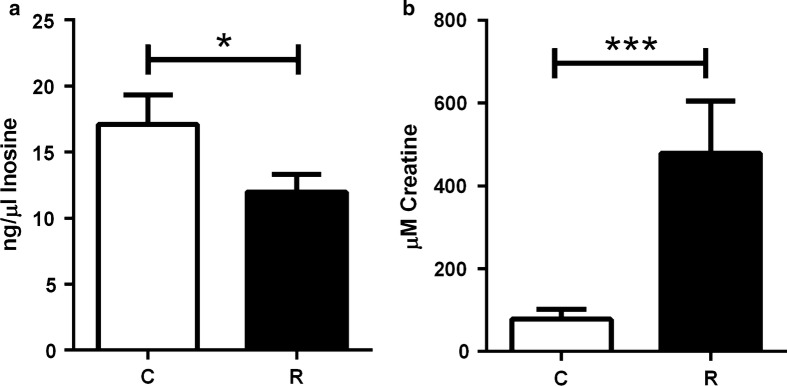
Inosine and creatine levels in serum change with *E. leei* infection. Serum concentration of **a** inosine (ng/µl) and **b** creatine (µM) in control (C, *n* = 8) and recipient (R, *n* = 20) fish from trials 1 and 2. Asterisks denote statistical significance at *P* < 0.05 (*) and *P* < 0.001 (***). Data are presented as mean + SEM (Mann–Whitney test; **a** *P* = 0.044, **b** *P* = 0.0004)

The application of the commercial ELISA kits for inosine and creatine showed significant changes in the serum of parasitized fish. The values of fish from trials 1 and 2 were merged to have a higher sample size and statistical robustness (C: *n* = 8; R: *n* = 20). Inosine was significantly decreased (Mann-Whitney U-test: *U*_(8)_ = 38, *Z* = 2.01, *P* = 0.045) (Fig. 6a), whereas creatine increased (Mann–Whitney U-test: *U*_(7)_ = 11, *Z* = − 3.53, *P* = 0.0004) (Fig. 6b) in parasitized fish.

## Discussion

The gastrointestinal (GI) tract acts as a barrier between the external and internal environments and thus the integrity of this barrier is crucial to maintain homeostasis. The barrier function of the gut is supported by epithelial cells, mucus, tight junction (TJ) and adherens junction (AJ) proteins [36]. The fish-parasite system used in the present study provides an excellent model to study the disruption of this barrier, as *E. leei* dwells in the paracellular space of the gut epithelial palisade. First of all, we have shown the functional disruption of the gut through the increased gut permeability and the decreased transepithelial resistance in parasitized fish. Secondly, we have demonstrated by IHC the decreased presence of some TJ proteins that are the building blocks of the gut barrier, especially claudin-3. Finally, we have outlined the utility of non-targeted serum metabolomics to detect marker metabolites of the disease condition and we have validated the use of creatine and inosine as disease markers of enteritis.

Epithelial permeability function has been assessed in mammals by *in vitro* or *ex vivo* methods such as transepithelial electrical resistance and *in vivo* tests such as transepithelial passage of different markers [22, 37, 38]. Intestinal mucosal barrier permeability is considered as an effective indicator of the integrity of the mucosal barrier. Experiments on intestinal barrier permeability in fish have been mainly based on *in vitro* and molecular studies such as gene expression studies [11, 13, 39, 40] and very few studies are available using *in vivo* markers [14, 41]. Among the *in vivo* methods, FITC-dextrans are primarily used for studying permeability and transport in tissues and cells, but to the best of our knowledge this is the first time that it is used in fish gut studies. Here, we chose a molecular size that allows studying the intestinal paracellular transport, as we hypothesised that the parasite location was altering it (either blocking or favouring). Indeed, what we found was a leaking effect, as the FITC-dextran was increased in the plasma of parasitized fish. Similarly, intestinal permeability was significantly elevated in different fish species after an infectious pancreatic necrosis virus (IPNV) challenge [42] and the paracellular permeability for Evans blue and D-lactate were significantly higher at both 24 and 72 h post-infection with *Aeromonas hydrophila* [14]. The leaking effect was confirmed by the decreased transepithelial resistance in parasitized intestines. These results agree with previous studies showing that *E. leei* disrupts intestinal water uptake, as a significant negative correlation between plasma chloride concentration and condition factor. Thus, a significantly higher osmolarity of plasma and major ion concentrations of the intestinal fluid were found in *E. leei*-infected tiger puffer (*Takifugu rubripes*) [43]. Some fish diets containing high levels of alternative vegetal protein sources may also induce digestive disturbances including diarrhoea-like conditions, indicating impaired gut permeability of water [44, 45]. Similarly, in GSB, some extreme vegetable diets impair Rt and this negative effect can be overcome when a butyrate additive is added [33]. Several human enteric protozoan parasites typically induce diarrhoea by a combination of different actions that alter gut integrity. For example, *Entamoeba hystolitica* degrades the protective mucus layers and evokes mucus hypersecretion. Its interaction with epithelial cells directly induces pro-inflammatory responses and later on perturbs the TJ proteins to stimulate water and ion secretion [46]. The diarrhoea induced by the intracellular parasite *Cryptosporidium parvum* is due to an increased paracellular permeability associated with decreased levels of several TJ and AJ proteins *in vitro* and also to the downregulation of genes related to TJs and AJs in response to the infection in *ex vivo* and *in vivo* mouse models [47]. Similarly, the reduction in the intestinal barrier function induced by *Giardia duodenalis* implicates disruptions of several TJ proteins [48].

The observed changes in permeability and Rt in the current fish-parasite model could also be due to the decreased presence of some TJ proteins in GSB parasitized intestines, as shown by IHC. TJs in enterocytes separate the intestinal lumen from the under-lying tissues, regulating the movement of ions and macromolecules, and thus maintaining the homeostasis. Claudins are essential components of TJs regulating paracellular solute transport. Claudins can alter or be altered by a number of signalling molecules/pathways. Abnormal expression and/or mislocalization of claudins are associated with many human and animal diseases [49]. Some studies have shown that the paracellular resistance of CLDN3-transfected monolayers was strongly elevated, causing an increase in transepithelial resistance. CLDN3 altered the TJ meshwork and sealed the paracellular pathway against the passage of small ions [50]. The downregulation of claudins at protein and gene level can be induced by different factors, including inflammation [51]. In teleost fish, at least 63 claudin genes have been described, but very little is known about their role in the GI tract physiology [52]. The abundance of claudins can vary spatially along the GI tract of teleosts and it progressively “tightens”, from the anterior to posterior part, thus preventing leakage of water back into the gut lumen [52–54]. Different dietary interventions have variable effects on fish intestinal TJs. Vitamin A deficiency decreased the mRNA levels of TJ complexes (several *cldns* and *tjp1*) in grass carp (*Ctenopharyngodon idella*) [55], dietary isoleucine decreased the expression of several *cldns* in Jian carp (*Cyprinus carpio* var. Jian) [56], dietary deoxynivalenol (a mycotoxin) also decreased the relative expression of markers for three TJ proteins in Atlantic salmon (*Salmo salar*) intestine [57], and some plant proteins induced significant alterations of the TJ signalling pathway in this same species [11]. By contrast, dietary stachyose increased the gene expression of *cldn3* and *tjp1* in turbot (*Scophthalmus maximus*) [58], and an olive oil bioactive extract increased *cldn3* expression in GSB [59], whereas some dietary interventions did not change the expression of *tjp1* in GSB [60].

The deleterious effects of pathogens on intestinal TJ integrity is poorly featured in fish, and initially determined by morphological changes [61–63]. More recently, the effect of pathogens on *cldn* transcript abundance in the intestine following viral and bacterial experimental infections has also been reported but with opposite trends. Claudin genes were significantly downregulated in the intestine of catfish (*Ictalurus punctatus*) at three hours post-infection with *Edwardsiella ictaluri*, the bacterial agent causing enteric septicemia [64]. Similarly, the expression of *tjp1* and several *cldns* was decreased in grass carp 72 hours after *Aeromonas hydrophila* infection [14]. On the other hand, following cyprinid herpesvirus 3 (CyHV-3) infection, mRNA encoding for several *cldns* signifi-cantly increased in the intestine of common carp (*Cyprinus carpio*) in conjunction with an upregulation of genes involved in the inflammatory response. It was proposed that alterations in *cldns* abundance may contribute to mechanisms that compensate for a possible disruption of proteins by nitric oxide produced during an immune response of the host to virus-induced tissue damage [65]. No information is available on the effect of fish parasites in intestinal TJs.

In the present study we did not observe a strong change in the intestinal immunolabelling of CDH1; however, its gene expression was significantly downregulated in severely *E. leei*-infected GSB [66]. Classical cadherins, such as E-cadherin (CDH1), are the major transmembrane proteins of AJ and initiate intercellular contacts through trans-pairing between cadherins on opposing cells. Formation of the AJ leads to assembly of the TJ, but E-cadherin is not required to maintain TJ organization [67]. Alterations of E-cadherin are associated with a variety of gastrointestinal disorders. In mammals, intestinal E-cadherin downregulation is usually observed in diseases characterized by high levels of pro-inflammatory molecules, such as inflammatory bowel disease [68, 69]. In fish, E-cadherin gene expression was modulated in the intestine of Atlantic salmon in response to an experimental diet that affected intestinal fluid permeability [44]. In previous studies in GSB, the intestinal gene expression of E-cadherin was also found to be modulated by some dietary interventions. In particular, it was significantly upregulated in GSB fed a diet low in fish meal and fish oil, and it was restored when sodium butyrate was added [33]. However, no changes were detected when fed with Next Enhance^®^150 [54] or with olive oil bioactive compounds [59], and a lower expression was found in the anterior intestine of fish fed DICOSAN or probiotics [70].

In any case, we cannot reject that the changes found in the intestinal barrier integrity could also be due to enterocyte apoptosis and necrosis or to the inflammatory response induced by the parasite, which have been described as acute/chronic in enteromyxosis [15, 71], or to changes in the intestinal mucus layer. In fact, *E. leei*-parasitized GSB have altered glycoprotein profile of the secreted intestinal mucus, bacterial adhesion to large-sized mucus glycoproteins is decreased [72], and important changes in goblet cell composition and distribution and intestinal mucin expression are found [73, 74]. These changes in the intestinal mucus can have a clear effect on the gut barrier, as epithelial TJs and the mucus layer cooperate to form a highly integrated barrier system that together limit access of luminal contents to the body. The capacity of the mucus to prevent abrasion and trap bacteria represents the first line of defence, while the paracellular TJ barrier prevents leakage of bacterial antigens from the lumen into the body [3].

Altered permeability may lead to impaired digestive functions and reduced fish growth [75], and arrested growth is one of the disease signs of this enteritis [76, 77]. In the present study, this was also evidenced by the differences in weight between R and C fish at the end of all trials. The loss of barrier function can also potentiate systemic absorption of pathogens and toxic molecules which has been shown to be associated with intestinal inflammation in mammals and fish [78, 79].

The untargeted metabolomics study of the serum showed significant changes in the profile of parasitized fish and the PLS-DA clearly separated parasitized fish from control ones into different clusters, confirming the stability and reproducibility of the LC-MS analysis. In previous studies, we have shown that this approach can detect differences in dietary interventions and the nutritional status of GSB [25, 26]. Metabolomics have been applied recently in several areas of aquaculture [27], including infectious fish diseases [24]. However, its application in fish parasitic diseases is very scarce, and only done thus far in naturally infected fish. In one of the few studies, in *Coilia nasus*, from the 391 annotated compounds, 65 metabolites were significantly regulated in Anisakid-infected groups, and the multivariate analyses of the serum metabolite profiles showed good separation between infected and non-infected samples [80], as in the present study. In a GC/MS study of a very similar enteric myxozoan disease, the PLS-DA of 53 metabolites showed three distinct groups according to their parasite load [81]. In *E. leei*-infected sera, the regulated metabolites were involved mainly in amino acid catabolism, fatty acid oxidation, nucleoside, lysophospholipid, vitamin and polyphenol metabolism. Similarly, in the above mentioned cases, the main pathways affected by the parasitic infection were amino acids and fatty acids [81] and amino acids, nucleotide derivatives, phospholipids, and immune-related metabolites [80].

In the present GSB metabolomic profile, some of the regulated compounds deserve special attention. Interestingly, two vitamins, biotin (vitamin B7) and pantothenic acid (vitamin B5) were more downregulated in severely infected GSB than in slightly infected animals. Biotin was also downregulated in short-term fasted fish [25], and we consider that the lowered levels of these vitamins could be due to the reduced nutrient availability reflecting the poor nutritional status of parasitized fish. Further studies are needed to determine the specific role of these vitamins on the pathophysiology of enteromyxosis and its possible therapeutic use, since several studies have shown the role of the intestinal biotin uptake system in the maintenance of mucosal integrity [82]. Biotin deficiency also induces active intestinal inflammation in mice similar to that observed in ulcerative colitis [82, 83] and leads to an array of pathological conditions in humans, including inflammatory bowel disease [84]. In addition, under biotin-deficient conditions, innate immune system cells produce increased levels of pro-inflammatory cytokines and Th1- and Th17-mediated proinflammatory responses in human CD4+ T lymphocytes [85]. Furthermore, both deficiency and excess of dietary pantothenic acid downregulate several *cldns*, *occludin* and *tjp1* mRNA levels in all intestinal segments of grass carp [86], and dietary deficiency of another vitamin (vitamin A) also impaired physical barrier functions associated with impaired antioxidant capacity, aggravated cell apoptosis and disrupted TJ complexes in the intestine of grass carp [55]. In contrast, another vitamin related compound, para-aminobenzoic acid (PABA), was increased in parasitized fish. PABA is an intermediate in the synthesis of the vitamin folate by bacteria, plants and fungi. Many bacteria, including those found in the human intestinal tract generate PABA. Humans lack the enzymes to convert PABA to folate, so require folate from dietary sources, such as green leafy vegetables, and rely on the intestinal microbiota. This also happens in fish, as Duncan et al. [87] demonstrated that intestinal microorganisms are a significant source of folic acid for channel catfish, and Kashiwada et al. [88] isolated folic acid-synthesizing bacteria from the intestine of common carp. Therefore, it is tempting to suggest that the intestinal alteration induced by the parasite could also induce changes in the intestinal microbiota of our fish, and therefore changes in the microorganisms capable of converting PABA to folate. Further research on microbial changes in the intestine of parasitized fish will help elucidate these changes.

Several carnitine-related compounds and two γ-glutamyl dipeptides were strongly increased in parasitized GSB (again, more in severely infected than in slightly infected animals). High circulating concentrations of γ-Glu-(Leu/Val/Ile) and five sub-products of l-carnitine were also found in the serum of fasted GSB [25]. These authors suggested that the increased levels of γ-glutamyl dipeptides were due to changes in the Meister’s glutamyl cycle, which has a key role in the recovery and delivery of cysteine in the body and transport of amino acids across cell membranes [89]. One of the key actors of this cycle is γ-glutamyl transferase (GGT), an enzyme that generates γ-glutamyl dipeptides by transferring the γ-glutamyl moiety from glutathione (GSH) to amino acids. Expression of GGT is essential in maintaining the cysteine levels in the body. Induction of GGT expression in response to redox stress provides the cell with access to additional cysteine, which becomes rate-limiting for intracellular GSH synthesis. Increased levels of plasma GGT were found in mice with viral infection [90], and in the liver and muscle of GSB fed diets with high levels of plant proteins [91]. This cycle could also be altered by changes in GSH. In fact, several glutamyl dipeptides have been used as biomarkers of human liver diseases because in healthy individuals the level of hepatic GSH is high and a small amount of GSH is biosynthesized. However, in patients with liver diseases, GSH is consumed to neutralize the generated ROS, which in turn leads to glutamyl cysteine synthetase (GCS) activation, resulting in the biosynthesis of GSH together with glutamyl dipeptides [92]. We can only speculate about this activation in the present study, but it is tempting to suggest it could also happen, as ROS are increased in parasitized GSB and a counteracting role of ROS was hypothesized when downregulated gene expression of *gpx-1* was found in the head kidney and intestine of parasitized GSB [76].

The increased levels of carnitine-related compounds in parasitized GSB are interpreted as increased mobilization of body fat stores, common in fasted individuals, exemplified by the loss of body weight in parasitized fish. Carnitine is actively transported into the cytosol to participate in the shuttling of activated long chain fatty acids into the mitochondria where β-oxidation takes place. During fasting and malnutrition, metabolic adaptations are triggered by PPARα (peroxisome proliferator-activated receptor alpha) to minimize the use of protein and carbohydrates as fuel to allow survival during long periods of energy deprivation and lipolysis pathways are engaged instead. Carnitine plays a critical role in energy balance across cell membranes and in energy metabolism of tissues that derive much of their energy from fatty acid oxidation such as cardiac and skeletal muscles [93]. In our case, the long-term infection also engaged protein catabolism in parasitized GSB, since different metabolites related to amino acid catabolism were highly increased, as is the case for oxoadipic acid (more than 4700% in highly parasitized fish), which is a key catabolite of the essential amino acids tryptophan and lysine.

The two selected metabolites (creatine and inosine) emerged as good markers to differentiate C and R fish. Creatine was significantly increased in proportion to the degree of infection in parasitized GSB, and also when the ELISA was performed in additional samples. Creatine is a nitrogenous organic acid, made from arginine, glycine and methionine. It is a key component of phosphocreatine, which works as a store for high energy phosphate in the muscle, as ATP is produced at the expense of ADP *via* the phosphocreatine shuttle and creatine kinase in active muscles. It is generally accepted that creatine increases as muscle protein is broken down and creatine levels are maintained by diet and endogenous synthesis. In fact, in humans, creatine amounts to more than 20% of the dietary intake of arginine [94]. The same happened for inosine, but with the opposite trend. Inosine, an endogenous purine nucleoside formed by the degradation of adenosine, is produced and released into the extracellular space during normal cell metabolism. Adenosine has a short half-life, whereas inosine has a much longer *in vivo* half-life. It was originally thought to have no biological effects. However, recent studies demonstrate that inosine has potent immunomodulatory and neuroprotective effects and increased inosine levels are present in various inflammatory states and heart conditions [95, 96]. We can only speculate about the meaning of the low levels found in parasitized GSB, which point to a dysfunction of purine metabolism. The first hypothesis is a decreased catabolism of adenosine, in an effort to maintain the fish energy homeostasis, due to the involvement of adenosine in ATP/ADP balance. The second would be the uptake of inosine by the parasite, as shown for parasitic protozoa that lack the enzymes required for *de novo* synthesis of purines and are therefore reliant upon the salvage of these compounds from the external environment [97]. Unfortunately, we do not have such information for *E. leei*, but recent genomic data of another myxozoan, *Thelohanellus kitauei*, seems to indicate that this parasite has lost the ATP-expensive pathways for *de novo* biosynthesis of inosine 50-phosphate and uridine 50-phosphate. Therefore, it must rely on salvage pathways as well [98]. If this is the case of *E. leei*, the possible therapeutic use of inosine against enteromyxosis is worth further investigation, since dietary inosine supplementation reduced the oxidative stress and improved intestinal health condition and immune response in several fish species [99, 100]. In fact, treatment with inosine compounds is currently being used for some human viral infections [101].

## Conclusions

**Fig. 7:**
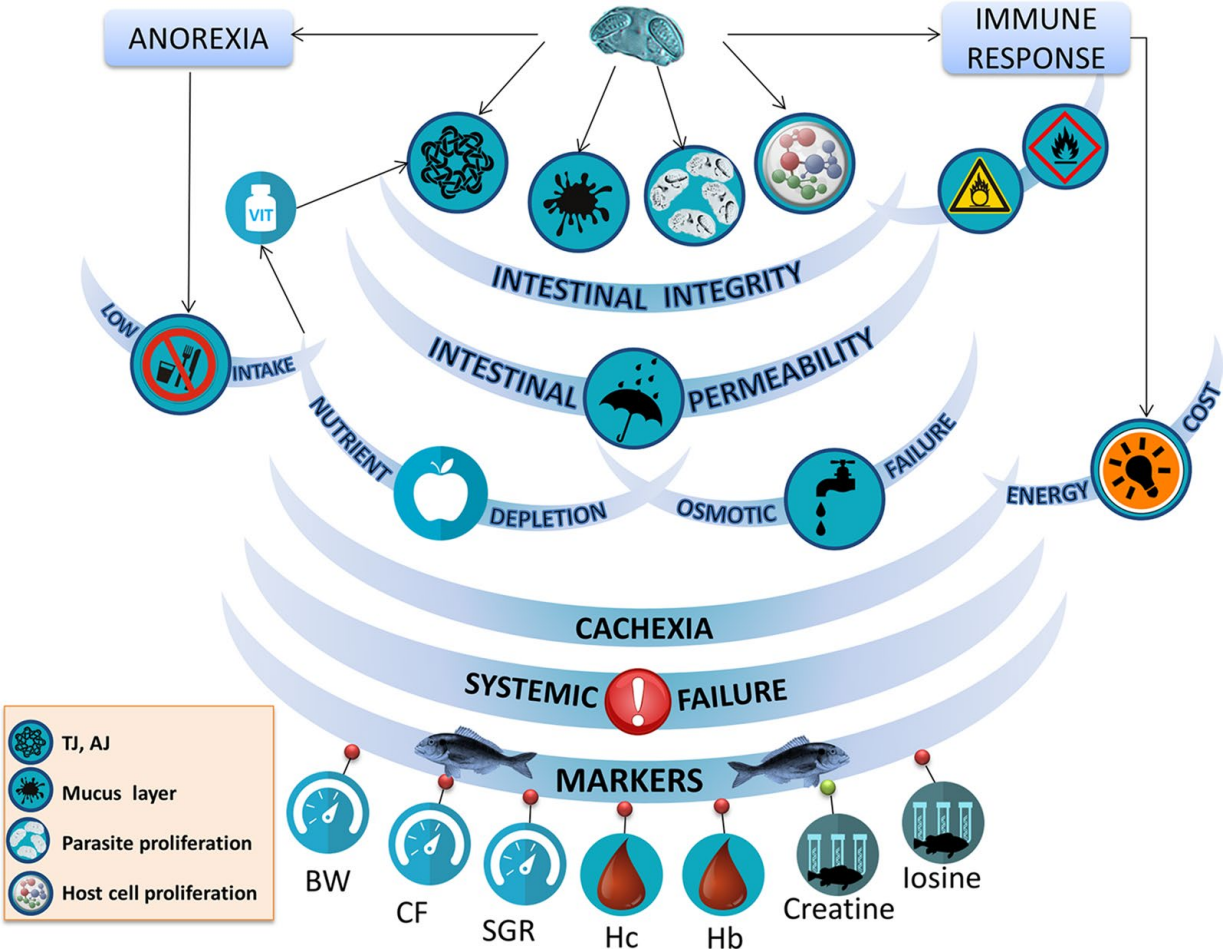
Proposed integrative model of the pathophysiology of *Enteromyxum leei* in the gilthead sea bream from the present study and previous published data [43, 71, 73, 74, 102, 103]. The parasite induces anorexia, immune responses and changes in intestinal integrity. Integrity is altered due to changes in tight junctions (TJ), adherent junctions (AJ), mucus layer, parasite proliferation and host cell proliferation. Intestinal integrity is also affected by vitamin deletion, oxidative stress and inflammation. These changes are translated into gut permeability dysfunction, which, together with decreased food intake, produce nutrient depletion and osmotic intestinal failure. All this together with the energy cost of mounting an immune response, invokes cachexia and finally systemic failure and the death of the fish. The disease indicators are related to growth retardation [body weight (BW), condition factor (CF) and specific growth rate (SGR)], anaemia [haemoglobin (Hb) and haematocrit (Hc)] and serum decrease of inosine and increase of creatine

To our knowledge, our results provide the first functional evidence of the disruption of the gut integrity by the fish parasite *Enteromyxum leei*. The clear decrease of the immunolabelling of several tight junction proteins along the intestine of parasitized fish leads to changes in the intercellular sealing, the selective diffusion barrier between epithelial cells and the prevention of the free passage of molecules and ions across the paracellular pathway. This was substantiated by the increased gut paracellular uptake and the decreased transepithelial resistance in infected animals, which showed a diarrheic profile. We have also demonstrated that parasitized fish have a distinct serum metabolomic profile, and that two metabolites (creatine and inosine) are good markers to differentiate parasitized and non-parasitized fish. The depletion of several metabolites involved in vitamin pathways opens the door to find future new palliative treatments. These results allow drawing a better picture of the complex interplay of the different factors involved in the pathophysiology of this disease, which are summarized in Fig. 7. The disruption of the intestinal integrity contributes to nutrient malabsorption, osmoregulatory failure and cachexia that eventually contribute to systemic organ failure.

## Supplementary information

**Additional file 1: Figure S1.** Orthogonal PLS-DA S-Plot of injected serum samples. Ions enhanced by enteritis are at the top-right and those decreased by enteritis are at the bottom-left. In red, ions of the selected compounds that were elucidated.

## Data Availability

All data generated by this study are included in the article and its additional file. Metabolomics data have been uploaded as MetaboLights study reference MTBLS1194 and are available at http://www.ebi.ac.uk/metabolights/MTBLS1194. MetaboLights is an open access repository for metabolomics studies [104].

## Abbreviations

AI: anterior intestinal segment
AJ: adherens junction
C: control group
CDH1: E-cadherin
CLDN-3: claudin-3
dpe: days post-exposure
dpi: days post-intubation
FITC: fluorescein isothyocianate
GC/MS: gas chromatography/mass spectrometry
GCS: glutamyl cysteine synthetase
GGT: γ-glutamyl transferase
GI: gastrointestinal
GSB: gilthead sea bream
GSH: glutathione
HILIC: hydrophilic interaction liquid chromatography
IHC: immunohistochemistry
Isc: short-circuit current
LC–MS: liquid chromatography–mass spectrometry
NL: non-lethal sampling
PABA: para-aminobenzoic acid
PI: posterior intestinal segment
PLS-DA: partial least-squares discriminant analysis
R: recipient group
ROS: reactive oxygen species
RP: reverse phase chromatography
Rt: epithelial resistance
TJs: tight junctions
TJP1: tight junction protein 1
VIP: variable importance in projection
