# Supplementary figures and images for: Disruption of gut integrity and permeability contributes to enteritis in a fish-parasite model: a story told from serum metabolomics
Source: Parasit Vectors. 2019 Oct 16;12:486. doi: 10.1186/s13071-019-3746-7 (PMC6796429; doi:10.1186/s13071-019-3746-7)

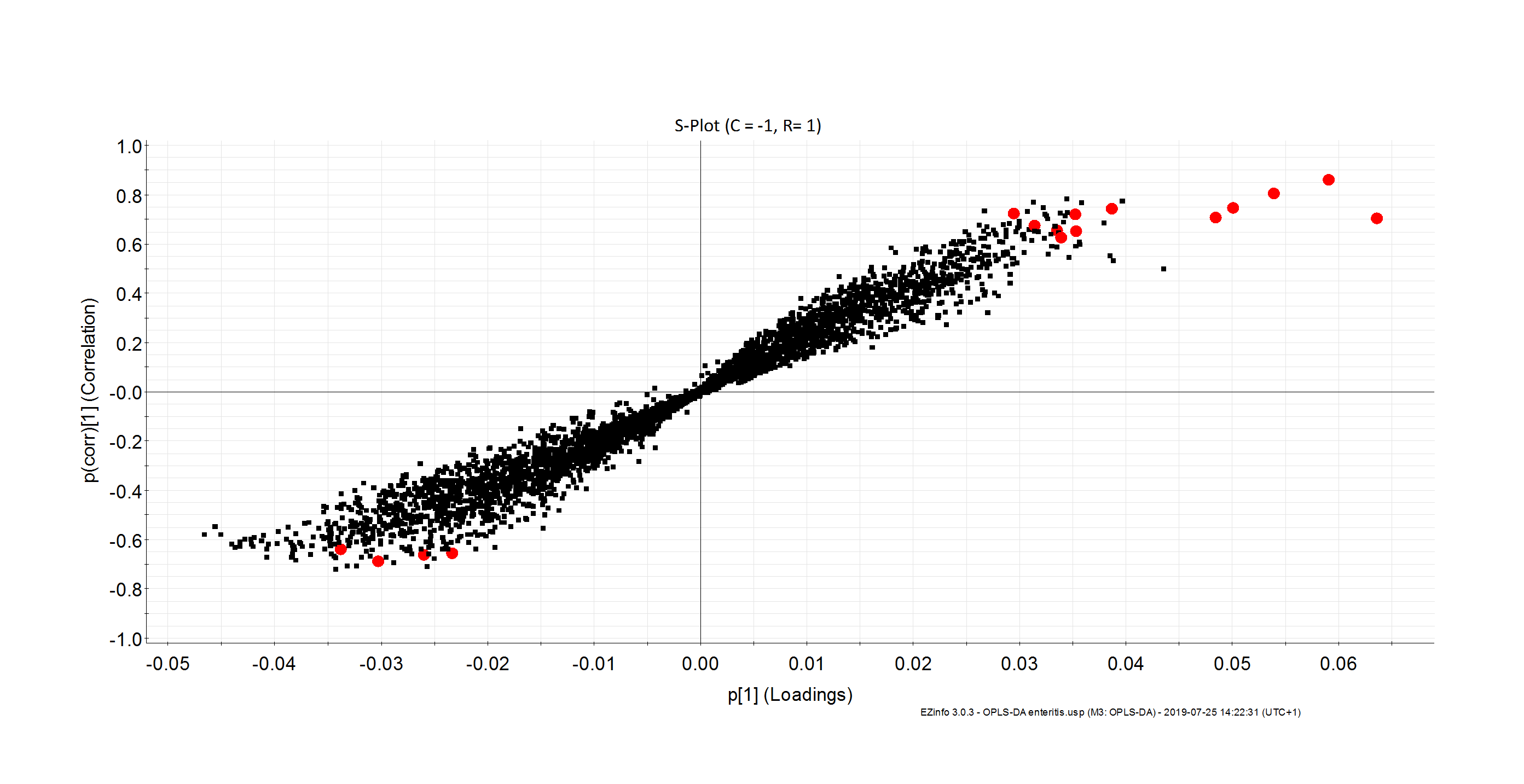

Supplement: Supplementary file 1 — Additional file 1: Figure S1. Orthogonal PLS-DA S-Plot of injected serum samples. Ions enhanced by enteritis are at the top-right and those decreased by enteritis are at the bottom-left. In red, ions of the selected compounds that were elucidated. [file 13071_2019_3746_MOESM1_ESM.tif]
